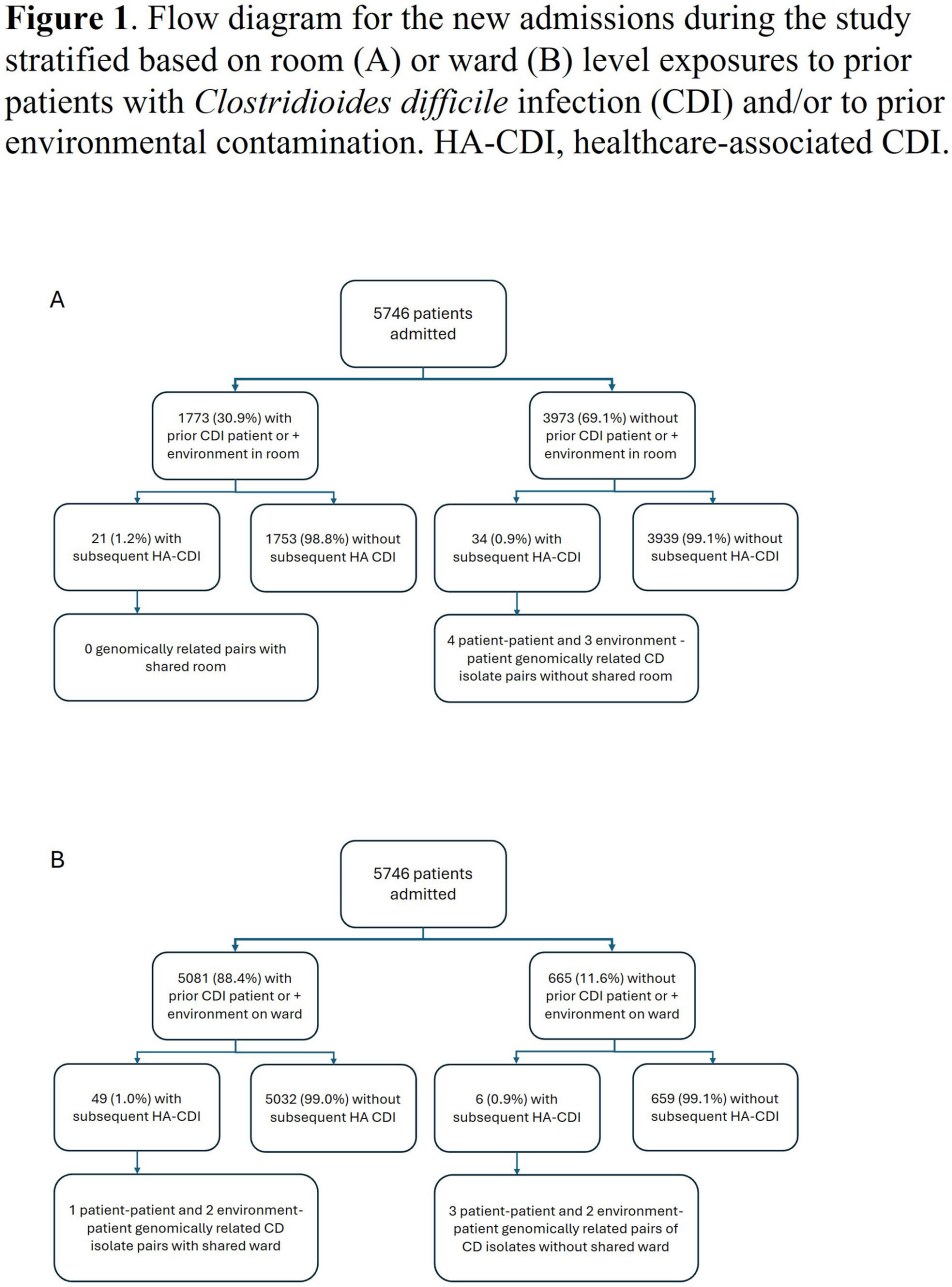# 14 Community deprivation and guideline concordant antibiotic prescribing for older adults in U.S. emergency departments, 2015–2024

**DOI:** 10.1017/ash.2026.10466

**Published:** 2026-06-23

**Authors:** Sarah Redmond, Maria Torres-Teran, Jennifer Cadnum, Claire Kaple, Munok Hwang, Hosoon Choi, Piyali Chatterjee, Chetan Jinadatha, Curtis Donskey

**Affiliations:** 1 Cleveland VA Medical Center; 2 VANEOHS; 3 Central Texas VA Research Foundation; 4 Central Texas Veterans Health Care System

## Abstract

**Background:** Admission to a room previously occupied by a patient with Clostridioides difficile infection (CDI) has been identified as a risk for CDI. However, previous studies have not included molecular typing to definitively link healthcare-associated CDI (HA-CDI) cases to prior room occupants or to residual spore contamination on surfaces. **Methods:** In an acute care hospital, we conducted a 1-year cohort study to determine the proportion of HA-CDI cases linked to prior room occupants with CDI or to room surfaces remaining contaminated after post-discharge cleaning and disinfection. Cultures were collected from post-discharge CDI and non-CDI rooms. Whole genome sequencing was used to determine relatedness of isolates. We calculated the percentage of HA-CDI cases infected with isolates genomically related to prior room or ward-level exposures. **Results:** Of 5,746 patients admitted, 22 had community-associated CDI and 55 were diagnosed with HA-CDI. C. difficile was recovered from 79 of 327 (21%) post-discharge rooms, including 14 of 36 (39%) CDI rooms and 83 of 287 (29%) non-CDI rooms. Of 1,773 patients with room-level exposure to prior CDI patients or contaminated surfaces, 21 (1%) developed HA-CDI, but none were infected with genomically-related isolates (Figure 1.A). Of 49 patients developing HA-CDI after ward-level exposures, 3 were infected with isolates genomically related to prior CDI patients or contaminated surfaces on the ward (1.B). **Conclusion:** Despite frequent exposure to rooms previously occupied by patients with CDI or contaminated with C. difficile, no HA-CDI cases were linked to prior room exposures based on whole genome sequencing.

**Figure:**